# Oil spill surface washing agents and chemical herders drive microbial community structure impacting biodegradation

**DOI:** 10.1128/aem.02334-24

**Published:** 2025-04-22

**Authors:** Kiara L. Lech, Devi Sundaravadivelu, Robert J. Grosser, Leah R. Trutschel, Nichole E. Brinkman, Robyn N. Conmy

**Affiliations:** 1 Office of Research and Development, Environmental Protection Agency 558795, Cincinnati, Ohio, USA; 2 Pegasus Technical Services, Inc., Cincinnati, Ohio, USA; 3 Oak Ridge Associated Universities 57870, Oak Ridge, Tennessee, USA; Universidad de los Andes, Bogotá, Colombia

**Keywords:** biodegradation, hydrocarbons, oil spill, bioremediation, surface washing agents, herders, spill treating agents, bacteria

## Abstract

Spill treating agents (STAs) may be authorized for use during an oil spill response; however, the impact of certain classes of agents on oil biodegradation is poorly understood. Microcosms comprising an oil-degrading microbial consortium were amended with weathered crude oil and treated with two STAs: a surface washing agent or chemical herder, alongside single-agent treatments. The microbial community readily degraded the STAs, evidenced by microbial growth and respiration. Carbon dioxide production was higher than expected in treatments containing oil and STA together, suggesting synergistic co-metabolism of otherwise recalcitrant compounds or dead-end metabolites. Within 14 days, n-alkanes and polycyclic aromatic hydrocarbons were biodegraded in oil-containing treatments by 90% and 57%, respectively. However, over the 40-day study, a fraction of higher molecular weight n-alkanes persisted in the oil treatment amended with the surface washing agent. The chemical herder initially confined the oil, limiting its bioavailability, but within 72 hours, the chemical herder was degraded, releasing oil hydrocarbons for subsequent biodegradation. Pronounced shifts in the microbial community were observed in all treatments with distinct differences between the total and active populations. Known contaminant-degrading families *Sphingomonadaceae* and *Rhodobacteraceae* were well represented in crude oil treatments, while groups such as *Gordonia*, *Rhodococcus*, and *Methylophaga* flourished in treatments containing STA. These findings demonstrate the outsized impact STAs have in shaping the activity and structure of the oil-degrading microbial community under laboratory conditions. However, uncertainty remains regarding the influence of these agents on oil biodegradation in real-world applications.

**IMPORTANCE:**

Spill treating agents offer oil spill responders alternative measures to reduce the overall impact of oil in the environment. Although the environmental implications of chemical dispersant use have been exhaustively studied under various conditions, this study aims to close knowledge gaps regarding lesser-known spill treating agents that may inhibit oil biodegradation. Results of this study demonstrated an impact on hydrocarbon degradation, highlighting significant differences in microbial community structure among the treatments. However, these agents were also readily biodegraded, potentially yielding limited influence on oil biodegradation in the environment. These findings broaden current understanding of how oil-degrading microbial communities may be affected by the use of spill treating agents, beyond chemical dispersants, ultimately aiding personnel tasked with operational decision-making during the critical stages of an oil spill response.

## INTRODUCTION

Biodegradation is an important natural process driving the fate of spilled oil in marine environments. Microbial communities exploit petroleum hydrocarbons as metabolic carbon sources because they are frequently exposed to anthropogenic inputs (e.g., oil spills, river run-off, and industry) and, to a greater extent, naturally occurring oil seeps (1). Temperature, the availability of nutrients and oxygen (or other terminal electron acceptors), the type of oil, and the degree of weathering all dictate the rate and extent of oil biodegradation (2–5).

During oil release incidents, spill treating agents (STAs) offer spill responders alternative mitigation tactics beyond mechanical recovery and *in situ* burning to remediate floating and submerged oil. In the United States, STAs that may be authorized for use are listed on the National Oil and Hazardous Substances Pollution Contingency Plan (NCP) Product Schedule (40 CFR § 300.900-920) in sub-categories based on intended function: chemical dispersants, surface washing agents, surface collecting agents (hereafter referred to as chemical herders), bioremediation agents, sorbents, and miscellaneous oil spill control agents. The magnitude and frequency of use among the categories vary widely and are difficult to ascertain. For example, dispersants or chemical herders may be used during a large (but rare), offshore oil spill, whereas surface washing agents would be utilized in near-shore or on-shore responses to both large and smaller, more frequent oil discharges.

In the wake of the unprecedented subsea application of chemical dispersants during the 2010 *Deepwater Horizon* oil spill (6), the effects of dispersants on naturally occurring oil biodegradation in marine environments have been heavily investigated and scrutinized. Some sources report dispersants enhancing oil biodegradation (7–10), while others cite their potential to suppress the biodegradation of petroleum hydrocarbons (11–13). Other STAs have not received the attention dispersants have, and deeper investigation is warranted regarding the biodegradability of oil treated with other STAs and the effects of these STAs on marine microbial communities. Two types of STAs are of particular interest: surface washing agents because of their routine application and chemical herders due to their potential benefits when used in burning operations in offshore remote locations, such as Arctic icy waters. Surface washing agents are used in shoreline oil removal and, more generally, decontaminating vessels and other response-related equipment. These agents are intended to lift the stranded oil from the substrate, and then the oil is washed into an adjacent body of water to be mechanically recovered (14). Currently, there are 62 surface washing agents listed on the NCP Product Schedule, with diverse modes of action and chemical composition. Conversely, only two chemical herders are listed. Herder suitability has been heavily tested at lab and field scales (15–17), but, to the authors’ knowledge, chemical herders have not been used operationally in the US since the addition to the NCP Product Schedule. Chemical herders are applied to the perimeter of oil slicks to corral the oil; the resultant thicker slick can be more effectively removed from the water surface via mechanical recovery or by increasing combustion efficiency during *in situ* burning (15).

This study aims to test the effects of two uninvestigated categories of STAs on oil biodegradation in a laboratory setting. The surface washing agent, CytoSol (CytoCulture International, Inc., Point Richmond, CA, USA), and chemical herder, ThickSlick 6535 (hereafter referred to as ThickSlick; EKL Leasing Company, West Seneca, NY, USA), were added to oil-degrading laboratory microcosms. Transformation and mineralization of weathered crude oil and STAs, changes in microbial biomass, and alterations of microbial community structure were measured and monitored for up to 48 days. Understanding the fate and effects of STAs at the laboratory scale helps inform emergency response and damage assessment efforts when making science-based decisions.

## MATERIALS AND METHODS

### Source oil and microbial inoculum

Alaskan North Slope (ANS) crude oil was obtained from Onta (https://onta.com/) and stored at ambient temperature. ANS was artificially weathered, adapted from King et al. (18), by sparging the oil with dry nitrogen flow at 25°C until mass loss stabilized at 8.7% after approximately 45 hours.

An oil-degrading microbial consortium isolated from a marine beach sample in Maine, USA, was sub-cultured and enriched on 3.4 mL of weathered ANS per liter of artificial seawater. Sterile artificial seawater, adapted from GP2 (19), contained, per liter, 21.03 g NaCl, 9.5 g MgCl_2_∙6H_2_O, 3.52 g Na_2_SO_4_, 2.89 g KNO_3_, 1.32 g CaCl_2_∙2H_2_O, 0.61 g KCl, 0.418 g K_2_HPO_4_, 0.17 g NaHCO_3_, 0.088 g KBr, 0.05 g FeCl_3_∙6H_2_O, 0.034 g Na_2_B_4_O_7_∙10H_2_O, and 0.02 g SrCl_2_∙6H_2_O. The pH was raised to 7.9 using sterile 1 N NaOH. The culture was shaken in the dark at 150 rpm at 20°C. Following 4 weeks of incubation, the cells were concentrated by centrifugation (8,000 × *g* for 10 min at 4°C) and washed in sterile artificial seawater. The cells were preserved in 25% glycerol in 5 mL aliquots and stored at −80°C. Prior to experimental setup, a portion of the cryopreserved microbial stock was thawed, washed twice, and brought up to volume with sterile artificial seawater. The cell culture was incubated on a shaker for 4 days and used as the microbial inoculum for the study.

### Experimental design

Microcosms were constructed in glass serum bottles (242 mL internal volume) containing artificial seawater as five treatments: ANS, ANS+CytoSol, ANS+ThickSlick, CytoSol, and ThickSlick. The inoculum from the enriched microbial consortium was added to the microcosms to achieve a 5% (vol/vol) cell concentration. In each applicable treatment, 80 µL ANS was added, and 40 µL STA was applied. With a total aqueous volume of 160 mL in the microcosms, the initial nominal concentrations of ANS, CytoSol, and ThickSlick were 450, 220, and 240 mg/L, respectively. Killed controls were constructed akin to the treatments described above but sterilized with sodium azide (500 mg/L) to account for potential abiotic loss or transformation of oil and STAs. Additional controls included the treatments minus the microbial inoculum and a treatment containing only microbial inoculum in artificial seawater. Treatments requiring sacrificial sampling were constructed in triplicate per sampling event. The serum bottles were sealed with polytetrafluoroethylene (PTFE)-lined stoppers and aluminum crimp caps. The bottles were placed horizontally on an orbital shaker set to 150 rpm at 20°C for up to 48 days. The microcosms were incubated in the dark to target heterotrophic activity, specifically avoiding any unintended activity or oxygen production via photosynthetic processes and precluding any impacts of oil photo-oxidation on oil biodegradation.

### Chemical analyses

Carbon dioxide and oxygen were monitored in the headspace of the microcosm bottles using gas chromatography. Using a gas-tight glass syringe, 100 µL of headspace was removed through the septum and directly injected into the gas chromatograph (GC; Agilent Technologies, Inc.; model 6890). Reported target analytes were within the lowest and highest concentration calibration standards. Agilent ChemStation software was used for instrument control and data acquisition.

Carbon dioxide production (as µmol CO_2_) was reported as a measure of biological respiration and mineralization of petroleum hydrocarbons and STAs. The percentage of oxygen was quantified to ensure aerobic conditions within the microcosm were maintained throughout the incubation period. Microcosms with headspace oxygen concentrations that fell, or were expected to fall, below 10% by volume were aseptically flushed with air to return oxygen concentrations to atmospheric levels. Headspace GC analysis was repeated immediately after microcosms were flushed to document new baseline gas concentrations.

Neat samples (10–20 mg) of weathered ANS, CytoSol, and ThickSlick were measured for total carbon (TC) and inorganic carbon (IC) using a Total Organic Carbon Analyzer (TOC-VCPH) with an SSM-5000A solid sample combustion module (Shimadzu Scientific Instruments, Somerset, NJ, USA) as per EPA SW-846 Method 9060A for the analysis of solid material for carbonaceous content (20). Samples were run in triplicate for both TC and IC, and the average of the difference was used to calculate the total organic carbon (TOC) in ANS and STAs (TC − IC = TOC, on a percent basis, wt/wt). The Shimadzu TOC-Control V software system achieved system control, data acquisition, and data processing.

For petroleum hydrocarbon analysis, microcosm samples (140 mL) were spiked with a labeled surrogate mix and sequentially extracted three times in a 250 mL separatory funnel using three 20 mL aliquots of methylene chloride (Optima grade, Fisher Scientific, Waltham, MA, USA). Each microcosm bottle was rinsed with 10 mL methylene chloride and added to the separatory funnel before extraction to ensure maximum recovery. Extracts were run through sodium sulfate and concentrated, under a stream of nitrogen at 29°C using a Zymark TurboVap II (Hopkinton, MA, USA), to a final volume of 5 mL. Analyte concentrations were quantified using a GC (Agilent Technologies 6890N) with 5975 series mass selective detector (MSD) and 7683 series autosampler, equipped with a DB-5 capillary column by J&W Scientific (30 m, 0.25 mm I.D., and 0.25 mm film thickness) following EPA Method 8270D (21). The samples were quantified against available standards (see the supplemental material) and reported as microgram analyte per liter aqueous sample. System control, data acquisition, and data processing were achieved with Agilent ChemStation. Concentrations of the detected, GC-MSD resolvable, individual alkanes and polycyclic aromatic hydrocarbons (PAHs) (Table S1) were normalized to hopane and summed to compute concentrations of total alkanes, alkanes C30-C35, total PAHs, and EPA priority PAHs.

Using the same sample extract described above, concentrations of total petroleum hydrocarbon (TPH; ANS-only treatment) and total extractable organic matter (TEOM; STA-only treatments) were measured by GC (Agilent Technologies 7890B) equipped with a flame ionization detector and 7693 autosampler following EPA Method 8015D (22). The TPH or TEOM, reported as milligrams per liter of aqueous sample, was quantified against the respective ANS or STA calibration curve for the corresponding treatment. The stock solution and six-point calibration dilution series were prepared in methylene chloride (Optima grade, Fisher Scientific). The peak quantification was performed using the total area under the curve, including the unresolved complex matter. System control, data acquisition, and data processing were achieved with Agilent OpenLAB CDS ChemStation.

For additional details of the chemical analyses described above, refer to the supplemental material.

### Microbial community composition

At each sampling point, 1.8 mL aqueous samples were removed from triplicate microcosms, immediately pelleted, snap-frozen in liquid nitrogen, and transferred to a −80°C freezer. Following the manufacturer’s extraction protocol, DNA and RNA were isolated from the pellets using the AllPrep Bacterial DNA/RNA/Protein Kit (Qiagen, Venlo, The Netherlands). DNA and RNA concentrations were, respectively, measured by Invitrogen Qubit dsDNA HS (high sensitivity) and Qubit RNA HS assay kits following the manufacturer’s instructions using a Qubit fluorometer (Thermo Fisher Scientific, Carlsbad, CA, USA).

Complementary DNA (cDNA) was synthesized from the purified RNA extracts using the Invitrogen SuperScript IV First-Strand synthesis kit using random hexamer primers (Thermo Fisher Scientific, Carlsbad, CA, USA). The reverse transcription time was extended to 50 min. Genomic DNA was removed prior to cDNA synthesis, and RNA was removed following the synthesis reaction, as per the manufacturer’s instructions.

The DNA extracts and newly synthesized cDNA (hereafter referred to as RNA) were sequenced using Illumina MiSeq (San Diego, CA, USA), targeting the V4 region of the 16S rRNA gene (primers 515F and 806R) at the Cincinnati Children’s Hospital Medical Center Genomics Sequencing Facility. The Mothur bioinformatics pipeline (version 1.48.0) was used for assembly and quality filtering of the reads following the standard operating procedure (23, 24). For taxonomic classification of reads, the Silva version 138.1 reference sequences adapted for Mothur were used (25). Samples were rarefied to contain 2,090 sequences, and any samples that contained less than this were removed from the sample pool.

Following analysis in Mothur, the sequences were processed in R (version 4.3.0) using the R package Phyloseq (version 1.46.0) for data analysis and figure generation (26). Taxonomic bar charts were generated by summing samples taken from replicate microcosms and then plotting the average relative abundance of the genera found within them. The top 10 genera with the highest average relative abundance across all DNA and RNA samples were then plotted to better visualize change over time and differences in the total and active communities. To compare the taxonomic composition of the various treatment groups, samples were assessed using Bray-Curtis dissimilarity and plotted using non-metric multidimensional scaling (NMDS). Permutational multivariate analysis of variance (PERMANOVA) was run using the Adonis2 function in the R Vegan package (version 2.6-4) to compare the centroids of the different treatment groups (27). A betadisper homogeneity of dispersion test was also conducted using the Vegan package to assess whether all treatment groups had a similar level of dispersion. This was followed by a series of pairwise Adonis tests using the pairwiseAdonis package (version 0.4.1) to determine which of the groups were significantly different from one another (28).

Finally, a differential abundance analysis was conducted using the R package DESeq2 (version 1.42) (29) to determine which of the most abundant genera were differentially enriched in treatments compared to the initial microbial inoculum. Nucleic acids (DNA and RNA) from the initial microbial inoculum were compared to the nucleic acids from the five treatment groups collected from days 10–40, excluding samples from the early transitional period (days 3–7) when the microbial communities underwent rapid transformation. Before analysis with DESeq2, the list of operational taxonomic units (OTUs) obtained from the sequencing data was filtered to include only the top 10 most abundant genera among all the samples. After comparing if the OTUs were differentially enriched in the treatment groups, results were filtered to an alpha value of 0.01, and the significant results were plotted in R.

Droplet digital polymerase chain reaction (ddPCR) was used to determine microbial growth throughout the experiment. Quantification of 16S rRNA genes in nucleic acid samples was performed using the QX200 Droplet Digital PCR System (BioRad, Hercules, CA, USA). The approximate number of prokaryotes per sample was determined by dividing the total number of molecules by 4.2, the average number of 16S rRNA gene copies per bacterial genome (30). Refer to the supplemental material for additional details on ddPCR methods.

## RESULTS AND DISCUSSION

### Respiration and microbial growth

**Fig 1:**
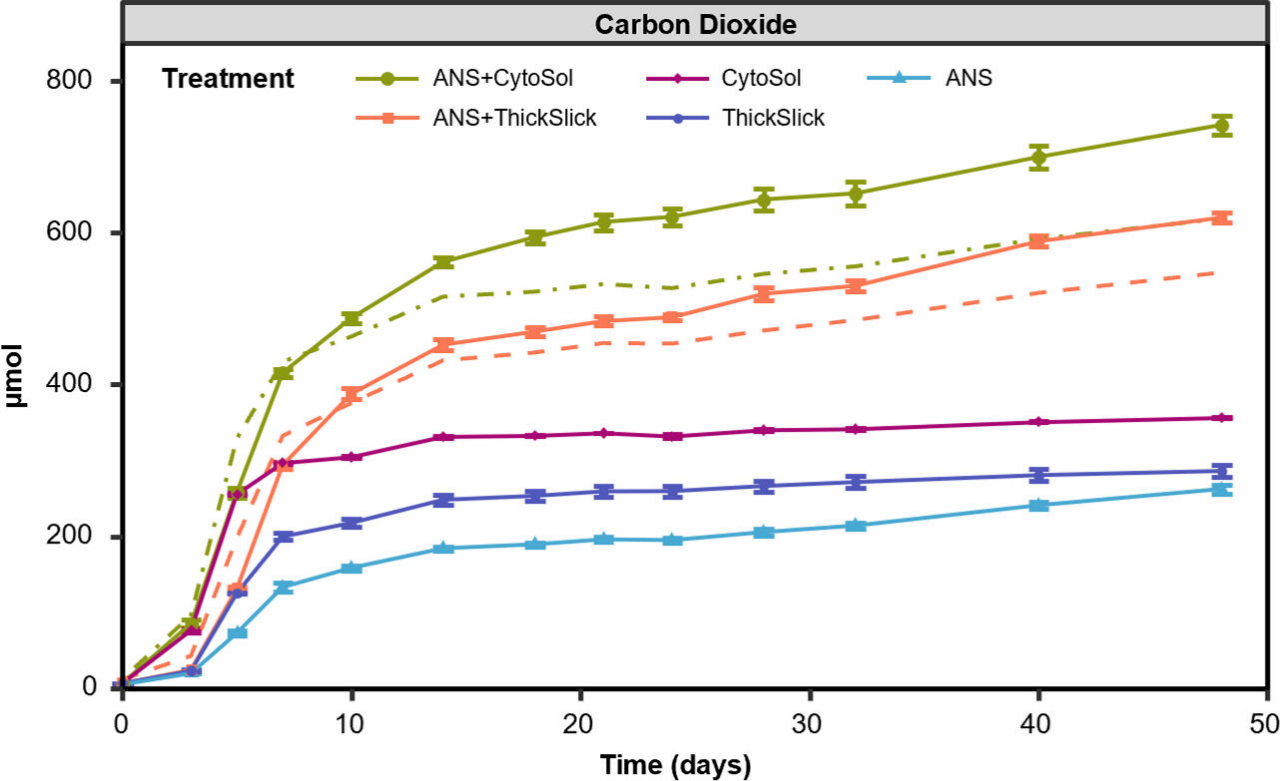
Cumulative carbon dioxide production over 48 days of incubation. Error bars represent ±1 standard error of triplicate microcosms. The dashed green and orange lines represent expected production in ANS+CytoSol and ANS+ThickSlick treatments, respectively. Expected carbon dioxide production was calculated by summing the production in individual treatments containing only one exogenous carbon source.

Microcosms consisted of an enriched oil-degrading microbial consortium in artificial seawater, supplemented with inorganic nutrients and amended with weathered crude oil and STAs as carbon sources. Due to compositional differences among STAs, monitoring biological respiration as an indicator of biodegradation was preferred over chemically identifying unique compounds that comprised each agent to track throughout the study. Moreover, the production of CO_2_ signified complete biodegradation (or mineralization) of the parent compounds to an end product. Over 48 days, total CO_2_ production in active treatments ranged from 260 µmol (ANS) to 740 µmol (ANS+CytoSol) CO_2_ (Fig. 1). Cytosol-containing microcosms produced more CO_2_ earlier than ANS and ThickSlick-containing treatments. As expected, the treatments comprising both ANS and STA produced the most CO_2_ during the study due to higher concentrations of added exogenous organic carbon. Respiration slowed considerably in all treatments following 2 weeks of incubation, particularly for the STA-alone treatments, suggesting that the available carbon in the STAs was quickly consumed and respired. However, cumulative CO_2_ production in ANS-containing treatments (ANS, ANS+CytoSol, and ANS+ThickSlick) continued to increase until the end of the study. All control treatments produced less than 20 µmol CO_2_ over the duration of the study.

**Fig 2:**
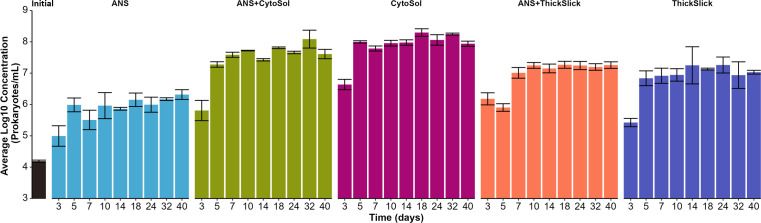
Microbial growth in each treatment compared to the initial microbial inoculum (black bar on far left), represented by the quantification of 16S rRNA gene in nucleic acid extracts. Values normalized by dividing by the average number of 16S rRNA gene copies per bacterial genome (30). Error bars represent ±1 standard deviation of the mean of triplicate samples.

Changes in microbial abundance were represented by the quantification of 16S rRNA gene copies across treatments. Microbial growth was observed in all treatments, with growth most limited in the ANS-only treatment (Fig. 2). The extent of microbial growth in treatments containing a mixture of ANS and STA (e.g., ANS+CytoSol) was similar to their STA-alone counterparts (e.g., CytoSol), except for ANS+ThickSlick before Day 7, which had delayed growth compared to ThickSlick-alone during the same time.

The STA-alone microcosms exhibited higher CO_2_ production compared to the ANS-alone treatments (Fig. 1), but only half the volume of STA was added to each microcosm compared to ANS (40 vs 80 µL, respectively). Moreover, the weathered ANS has higher TOC (85%) than both CytoSol and ThickSlick (78% and 64% TOC, respectively). This difference in CO_2_ production suggested that the two STAs were highly biodegradable under these experimental conditions and may have had a greater impact on microbial growth and respiration than ANS. CytoSol is comprised predominantly of long-chain fatty acid methyl esters (FAMEs) (31, 32). ThickSlick is made of 65% sorbitan monolaurate (Span 20) and 35% 2-ethyl butanol (17). These compound classes are known to be biodegradable (33–36), and the microbial consortium used in this study exploited these STAs as growth substrates. A previous study conducted by Fritt-Rasmussen et al. (37) showed that ThickSlick rapidly biodegraded within the first 7 days of incubation in seawater at 2°C, but Siltech OP-40, the only other chemical herder listed on the NCP Product Schedule, proved recalcitrant. A synergistic interaction occurred in ANS+STA treatments, between the agents, oil, and/or the resident microbial community, as indicated by greater CO_2_ production than the summed CO_2_ production in STA- and ANS-alone treatments, i.e., ANS+CytoSol [CO_2_] > ANS [CO_2_] + CytoSol [CO_2_]. Respiration in ANS+STA microcosms was expected to be the sum of CO_2_ production in individual treatments (dashed lines; Fig. 1). However, the observed CO_2_ production was about 17% and 12% higher than expected in ANS+CytoSol and ANS+ThickSlick, respectively (Fig. 1). This higher-than-expected CO_2_ production may be attributable to co-metabolism due to the addition of STAs as secondary substrates, similar to the results in Pasqualino et al. (38), demonstrating the synergistic effects of biodiesel (aka. FAMEs) on the biodegradation of diesel fuel. Crude oil is a complex mixture of hydrocarbons, a majority of which are not resolvable by GC-MSD, but some recalcitrant oil compounds may be susceptible to biodegradation via co-metabolism.

### Biodegradation of petroleum hydrocarbons

**Fig 3:**
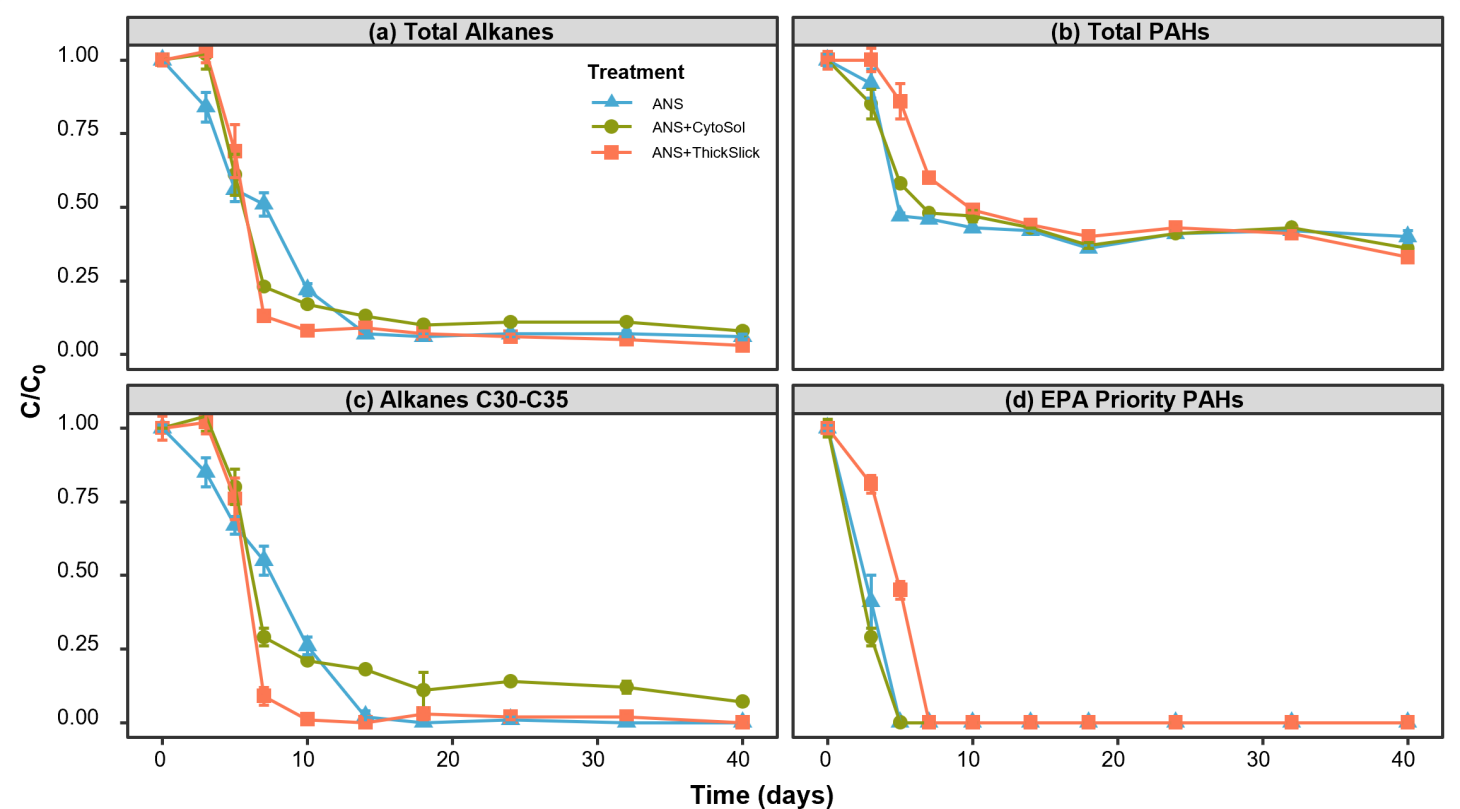
Aerobic biodegradation of oil hydrocarbons over 40 days. Individual analytes normalized to hopane and starting concentration. Error bars represent ±1 standard error of triplicate microcosms. Compounds that comprise (**a**) total alkanes, (**b**) total PAHs, (**c**) alkanes C30-C35, and (**d**) EPA Priority PAHs can be found in Table S1.

Total alkane and PAH concentrations were reduced, on average, across ANS-containing treatments by 90% and 57%, respectively, by day 14 (Fig. 3a and b). Adding STAs to microcosms induced a short lag in alkane degradation in the first 3 days of incubation. However, alkane degradation was faster in ANS+CytoSol and ANS+ThickSlick from day 3 to day 10 compared to the ANS-only treatment (Fig. 3a and c), suggesting STAs stimulated alkane biodegradation during this time, corresponding with periods of high CO_2_ production (Fig. 1). Higher molecular weight n-alkanes (C30-C35) were nearly consumed by day 14 in the ANS and ANS+ThickSlick treatments. However, 18% remained in ANS+CytoSol and gradually declined to a residual 7% by the final sampling time point (Fig. 3c). The degradation of PAHs was not impacted by the addition of CytoSol, as no lag period was observed, and the total mass loss of PAHs was comparable to that of ANS alone (Fig. 3b). However, like the alkanes, PAH degradation was delayed in ANS+ThickSlick in the first 72 hours. ThickSlick, as a chemical herding agent, is intended to increase the thickness of an oil slick. The addition of ThickSlick likely decreased the surface area of the oil droplets in the microcosms, slowing the dissolution and bioavailability of oil hydrocarbons. Hansen et al. (39) found that during the preparation of oil toxicity test solutions under low energy conditions, ThickSlick was able to maintain a contracted oil slick for at least 72 hours, and the authors reported a decrease in dissolved PAH concentrations in the aqueous phase during that time, compared to oil-only test solutions. The study presented here is distinctly different in test conditions and research objectives, but the data suggested a similar trend in the ANS+ThickSlick treatment. Although an average of 36% of total resolvable PAHs remained within the treatments by the study end, EPA’s High Priority Pollutant PAHs (Table S1), a standardized set of hydrocarbons often used for risk assessment, were below detection by day 5 in ANS and ANS+CytoSol, and by the following sampling time point (Day 7) in ANS+ThickSlick (Fig. 3d). However, it is important to note that across all the oil-containing treatments, the EPA’s High Priority Pollutant PAHs comprised only 4% of the initial total PAH concentration (Fig. S1), with many of the individual analytes having starting concentrations below the method reporting limit. In addition, alkylated 3- and 4-ring PAHs, many of whose parent compounds reside on the EPA Priority Pollutant list, dominate the residual fraction of resolvable PAHs, representing on average 92% of the recalcitrant PAHs at the conclusion of the study (Fig. S1). Studies have shown that the alkylated homologs of some PAHs are in higher concentration in crude oil, are frequently more persistent in the environment, and impart greater toxicity to aquatic biota than the unsubstituted parent PAHs (40–44).

Total petroleum hydrocarbon concentrations were monitored in the ANS-alone treatment, exhibiting a 42% reduction by day 14 (Fig. S2). A similar measure of total extractable (and combustible) organic compounds was evaluated in the STA-alone microcosms using the same analytical method as for TPH. By day 5, TEOMs were 100% and 93% consumed in the CytoSol and ThickSlick treatments, respectively (Fig. S2). It is hypothesized that the two STAs delayed the biodegradation of oil hydrocarbons via dissimilar mechanisms. ThickSlick, as mentioned above, may have reduced the bioavailability of oil alkanes and PAHs but was rapidly degraded, thus releasing the ANS for subsequent dissolution and microbial attack. Alternatively, in ANS+CytoSol, the presence of easily degradable, partially oxidized, long-chain fatty acid methyl esters that comprise CytoSol may have been the preferred substrate over the alkanes present in the weathered ANS (45). Once the components in CytoSol were exhausted, the microbial community shifted to alkanes in the crude oil as a carbon source (Fig. 3a and c).

### Microbial community composition

**Fig 4:**
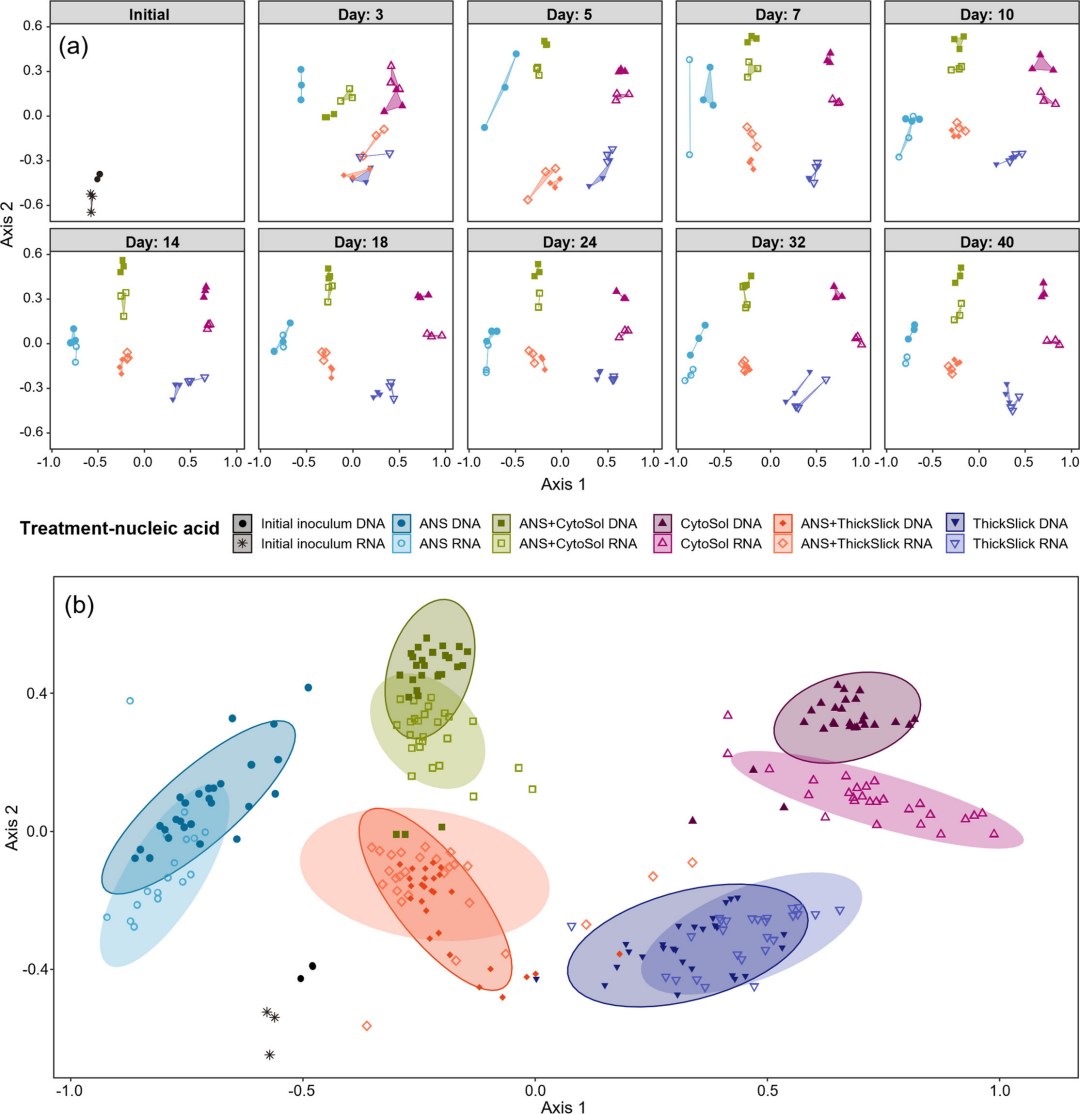
Nonmetric multidimensional scaling of Bray-Curtis distances comparing microbial community composition (**a**) delineated by sample day, and (**b**) as a single plot by treatment. Treatments are differentiated by both color and shape. Nucleic acid type is indicated by whether the shape is filled, where RNA is open and DNA is filled. RNA samples are absent from ANS on days 3 and 5 due to low yields during extraction.

**Fig 5:**
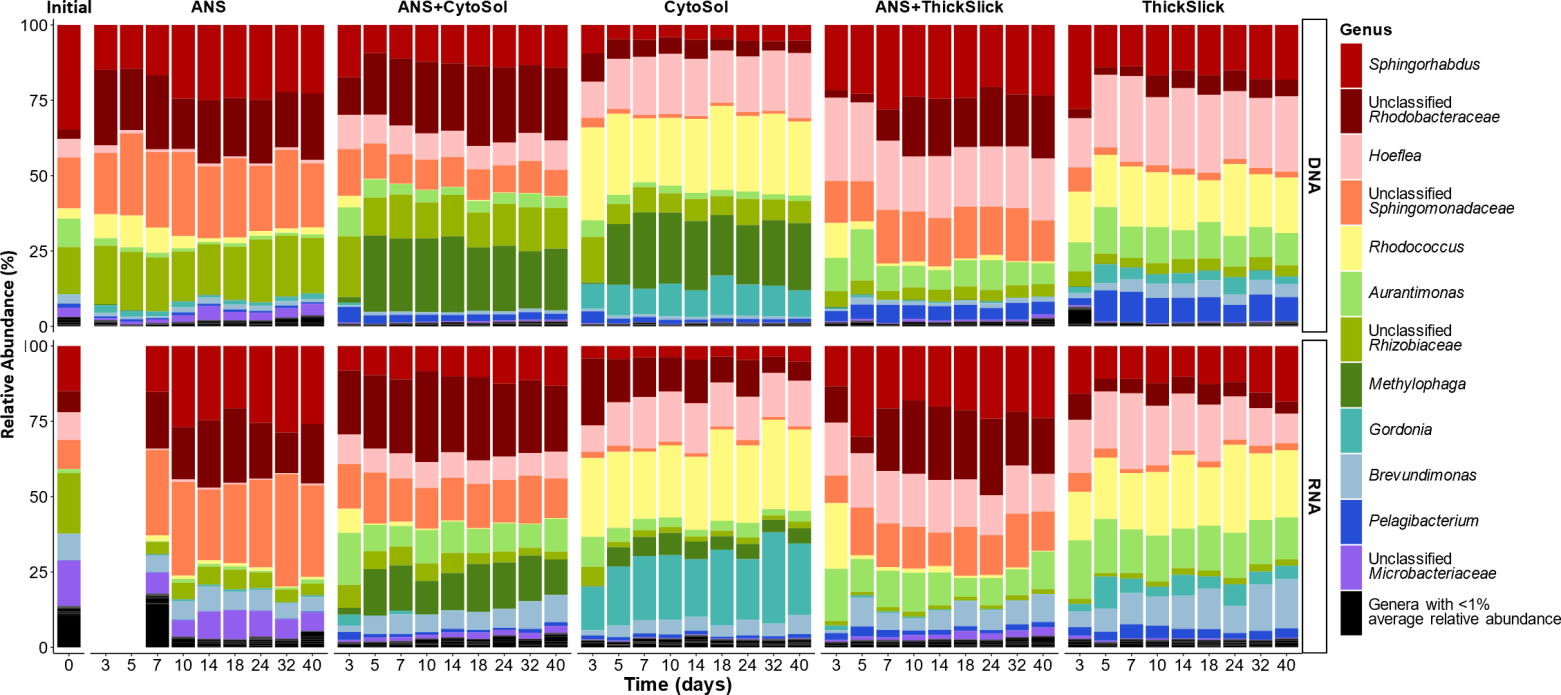
Taxonomic bar charts showing compositional changes within the total (DNA) and active (RNA) microbial communities within each treatment over time. Each bar represents the average relative abundance as determined from triplicate microcosms. Community composition is represented at the genus level, with genera having an average relative abundance of 1% or greater across all samples identified in the legend. RNA samples are absent from ANS on days 3 and 5 due to low yields during extraction.

The resident total and active microbial communities shifted away from the initial microbial inoculum composition as a function of time and treatment (Fig. 4). Amplicon sequencing of the 16S rRNA gene was used to characterize the total microbial community, whereas sequencing 16S rRNA gene transcripts (extracted RNA converted to cDNA) only represented the metabolically active microbial community. Transformation of the microbial community was immediate and began to diverge across treatments by day 3 (the first sampling time point following the study start). Distinct microbial communities were quickly established and appeared to stabilize in all treatments by day 10 (Fig. 4a and 5). As expected, the RNA fraction was less stable than the DNA fraction, with the active community rapidly responding to fluctuating conditions and resource availability over time. *Hoeflea*, *Gordonia*, and *Brevundimonas* illustrated the modulating active community, particularly in the STA-alone treatments (Fig. S3). These modest fluctuations were less likely to be captured when evaluating the total resident community alone.

The treatment groups all formed distinct, homogeneously dispersed clusters. The betadisper homogeneity of dispersion test indicated that the dispersion was not significantly different between treatments (DNA: *P* = 0.447; RNA: *P* = 0.538), and the initial comparison via PERMANOVA revealed that one or more of the groups significantly differed from the other groups when assessing total and active communities (DNA: *R*^2^ = 0.86, *F* = 165.18, and *P* = 0.001; RNA: *R*^2^ = 0.84, *F* = 125.18, and *P* = 0.001). Pairwise PERMANOVA (similarly parsed out by nucleic acid type) revealed that the centroids of each treatment group were significantly different from every other group (Table 1), suggesting that STAs appreciably altered microbial community composition and structure. The treatments containing both ANS and STA fell neatly between their respective single-component counterparts (Fig. 4b). Given the dissimilar but readily biodegradable compositions of CytoSol and ThickSlick (Fig. 1; Fig. S2), the marked differences in microbial communities among treatments could be anticipated and were well-supported.

**TABLE 1:** Pairwise Adonis comparisons of Bray-Curtis distances between treatments^*a*^

Treatment pair	DNA			RNA		
	*F* statistic	*R* ^2^	Adjusted *P* value	*F* statistic	*R* ^2^	Adjusted *P* value
ANS vs ANS+CytoSol	107.2	0.67	0.001	99.6	0.71	0.001
ANS vs ANS+ThickSlick	126.3	0.71	0.001	59.0	0.58	0.001
ANS vs CytoSol	387.0	0.88	0.001	304.7	0.88	0.001
ANS vs ThickSlick	301.5	0.85	0.001	261.3	0.86	0.001
ANS+CytoSol vs ANS+ThickSlick	129.0	0.71	0.001	42.3	0.45	0.001
ANS+CytoSol vs CytoSol	177.4	0.77	0.001	229.3	0.82	0.001
ANS+CytoSol vs ThickSlick	253.8	0.83	0.001	195.1	0.80	0.001
ANS+ThickSlick vs CytoSol	282.6	0.84	0.001	176.8	0.77	0.001
ANS+ThickSlick vs ThickSlick	85.0	0.62	0.001	83.4	0.62	0.001
CytoSol vs ThickSlick	118.1	0.69	0.001	90.0	0.64	0.001

^*a*^ Treatment pairs are composed of either DNA or RNA samples from all days collected. To account for multiple comparisons, the *P* value was adjusted using the Benjamini-Hochberg method.

PERMANOVA and subsequent pairwise PERMANOVA tests on all treatments and nucleic acid types revealed that the metabolically active microbial communities were significantly different from the total resident communities (*R*^2^ = 0.86, *F* = 143.38, and *P* = 0.001) yet maintained similar levels of dispersion (*P* = 0.403) (Table S2). Although the locations of centroids were significantly different, the clusters of resident and active communities for each treatment resided relatively close to each other and were visually distinct from the other treatment groups (Fig. 4b).

**Fig 6:**
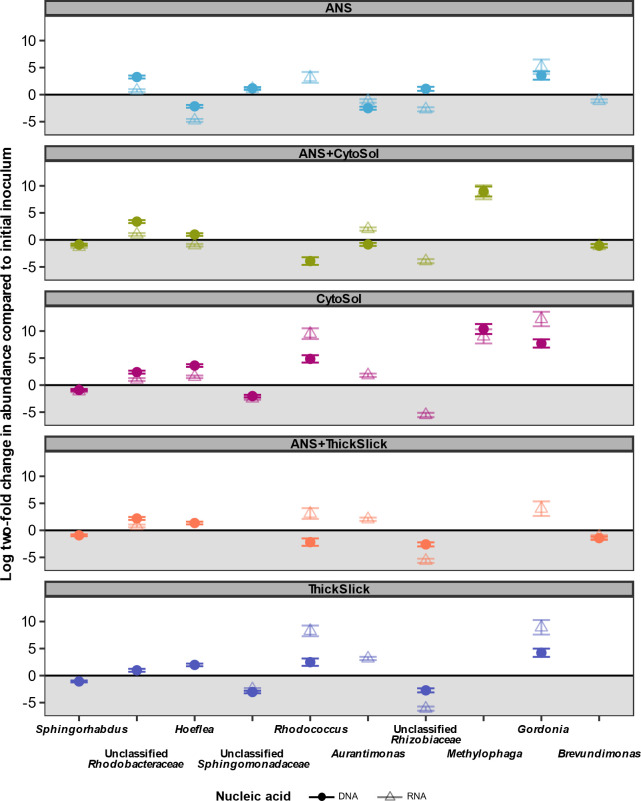
Differential abundance analysis using DESeq2 showing the differential enrichment of the top 10 most abundant genera across all treatments and nucleic acid types compared to the initial microbial inoculum. DNA and RNA samples from days 10–40 were used in comparison to the initial microbial inoculum. Taxa with points above the 0 line are significantly more enriched (*P*-adjusted value < 0.01) in the later part of incubation, while taxa with points below the 0 line, in the shaded region, are significantly more enriched in the initial microbial inoculum. A taxon with no data point in the plot is present in both the treatment and initial microbial inoculum but is not significantly enriched in either. Error bars represent ±1 of the Log2 fold change standard error of triplicate samples.

The shifting microbial communities in each treatment were characterized by enrichment and displacement of taxa originating from the oil-degrading inoculum (Fig. 5). Differential abundance analysis showed the statistical significance (*P* < 0.01) of differences in the observed (post-sequencing) absolute abundances of individual taxa between the initial microbial inoculum and the microbial communities established following incubation under the different treatment conditions (Fig. 6). Within the total microbial community (DNA-based analysis), *Sphingorhabdus* was the most abundant genus in the starting microbial inoculum. The relative abundance of this genus declined in every treatment (Fig. 5), with a significant decline in all treatments but ANS-alone (Fig. 6). Conversely, in the active community (RNA-based analysis), this population was less prominent in the starting microbial inoculum (Fig. 5), and only treatments containing CytoSol exhibited a significant reduction of *Sphingorhabdus* (Fig. 6). Most *Sphingorhabdus* strains were formerly classified as *Sphingopyxis*, a genus known for degrading various environmental contaminants (46, 47). Unclassified members of the family *Sphingomonadaceae* were found in abundance within the initial microbial inoculum and in treatments containing ANS, but their prevalence diminished in STA-only treatments (Fig. 5 and 6). The hydrocarbon-degrading members of this family are particularly known for degrading PAHs (48); the absence of PAHs in the STAs may explain the drop in abundance of the unclassified *Sphingomonadaceae* genera in the treatments that do not contain crude oil.

In ANS-containing treatments, the relative abundances of unclassified members of the family *Rhodobacteraceae* were enriched seven- and threefold in the total and active microbial communities, respectively, compared to the starting microbial inoculum (Fig. 5). *Rhodobacteraceae* are metabolically versatile and are prominent marine heterotrophs often found in oil-polluted waters (49, 50)*. Rhodobacteraceae* increased across all treatments, but in STA-alone treatments, the magnitude of growth was modest compared to other groups such as *Gordonia*, *Rhodococcus*, and *Methylophaga* (Fig. 6).

*Gordonia* was enriched within the CytoSol and ThickSlick (STA-alone) treatments (Fig. 5 and 6). *Gordonia* is found across habitats and various contaminated environments due to its diverse metabolic capabilities, and it is often touted for potential application in bioremediation and biotechnology (51, 52). This genus has been shown to degrade straight and branched alkanes, aromatic hydrocarbons, including anthracene, naphthalene, phenanthrene, pyrene, and benzene, as well as heterocyclic compounds and 2-butoxyethanol, the main component of the dispersant Corexit EC9527A used during the *Deepwater Horizon* oil spill response (51–55). *Rhodococcus*, a close relative of *Gordonia* (Table S3), was similarly enriched within the CytoSol and ThickSlick treatments (Fig. 6). This genus includes several species capable of degrading benzene, toluene, ethylbenzene, and xylene compounds, high molecular weight PAHs, medium and long-chain alkanes, and C1-12 alcohols (33, 56–58). Owing to their metabolic plasticity, these taxa may have been able to quickly pivot to consuming the newly introduced STAs. Relative to crude oil, these STAs are much simpler mixtures of carbon substrate, potentially conferring competitive advantage to a narrower set of taxa in the STA-only treatments. For example, *Gordonia* and *Rhodococcus* may have readily consumed the fatty acid methyl esters in CytoSol, which promoted their population growth so that they quickly became the most abundant genera within the active microbial community in the CytoSol-only treatment (Fig. 5).

As one of the top 10 most abundant genera across the treatments, *Methylophaga* was very significantly enriched, but only in the CytoSol and ANS+CytoSol treatments (Fig. 6). *Methylophaga* is a methylotrophic genus known to primarily degrade C1 compounds such as methanol and methylamine (59). In oil-contaminated environments, this group was hypothesized to operate as a secondary degrader of C1 compounds (60); however, an isolated *Methylophaga* sp*.* has been grown on n-hexadecane as a sole carbon and energy source (61). In the current study, the metabolic role of *Methylophaga* was not identified, but it is evident that the addition of Cytosol significantly favored the prevalence and activity of this population (Fig. 6).

STAs also selected for *Hoeflea* and *Aurantimonas*, members of the *Rhizobiaceae* family (Fig. 5). *Hoeflea* has previously been isolated from marine environments, including *Hoeflea olei,* which was isolated from diesel-oil contaminated water and found to degrade a wide range of diesel hydrocarbons as well as other simpler carbon substrates (62)*. Aurantimonas* has been identified in hydrocarbon-contaminated environments and has been shown to oxidize manganese in marine environments (63, 64).

Changes in abundance of specific taxa in total (DNA based) and active (RNA based) microbial communities trended together in each treatment. However, the magnitudes of these shifts were different (Fig. 5 and 6). In CytoSol-containing treatments, these community differences illustrated conflicting findings between the DNA and RNA fractions. For example, *Methylophaga* and *Rhizobiaceae* appeared to be overrepresented in the total community compared to their proportions in the active subset of the microbiome. Conversely, *Gordonia* dominance was underrepresented in the DNA fraction, with the difference in the relative abundance between the DNA and RNA fractions being about 12% (Fig. 5). *Methylophaga* had a greater increase in the DNA-based fraction than *Gordonia*, but the RNA fraction revealed the opposite pattern, showing *Methylophaga* with less metabolic activity compared to *Gordonia* (Fig. 6). A significant decrease in *Rhizobiaceae* was only observed in the active community, and no significant decline was observed in the total community (Fig. 6). These divergences were again illustrated in the NMDS, showing the least overlap in total (DNA) and active (RNA) populations in the CytoSol-only treatment compared to the other treatments (Fig. 4). This shift is likely attributed to the active community’s rapid response to the availability of CytoSol as a substrate, inducing the greatest change in microbial activity, as demonstrated by CO_2_ production and microbial growth (Fig. 1 and 2).

A similar study by Techtmann et al. (65) assessing changes in total and active communities in oil-degrading microcosms treated with chemical dispersant also showed substantial variations between the two populations. The microbial diversity and dominance of some members within the microbiome may be misinterpreted or overstated by exclusively targeting the 16S rRNA gene, which includes both metabolically active and dormant microorganisms. Sequencing 16S rRNA gene transcript (RNA-based analysis) concurrently with the 16S rRNA gene (DNA-based analysis) provides a more comprehensive understanding of the ecology and dynamics of contaminant-degrading microbial communities. Likewise, assessing shifts in the microbial community in terms of the relative and absolute abundances provides confidence that changes are both real and meaningful, tempering false inferences and yielding improved cross-study comparisons, which are often precluded by differences in experimental and technical methodologies.

The microbial consortium used in this study was enriched on oil with excess nutrients, potentially giving an advantage to oil-degrading microorganisms over microorganisms that may only be able to degrade CytoSol or ThickSlick alone. The impact of these STAs on oil biodegradation may be more substantial under conditions where the native microbial community is not previously exposed to crude oil. The addition of CytoSol slowed the degradation of higher molecular weight alkanes, potentially due to substrate preference. A more diverse and abundant microbial inoculum may be available *in situ*, where competition for resources and extent of biodegradation may be considerably different than those described here. ThickSlick temporarily delayed PAH degradation. However, this chemical herder was applied at a ratio of 1:2 with ANS, and it may have completely encapsulated the oil droplets, slowing dissolution rates in the microcosms. This study targeted the maximum conceivable ratio likely to be observed in the environment, intending to mimic concentrations found at the chemical herder-oil slick interface. In a real-world scenario, the relative volume of chemical herder to oil is much smaller, as chemical herder would be dispensed only around the perimeter of an oil slick with a resultant ratio somewhere between 1:500 and 1:1,000, across the contaminated area (39). Depending on the ratios in residual contamination, PAH degradation may be slightly hindered *in situ*, although it is improbable that ThickSlick would have any measurable impact on overall PAH degradation.

Two oil spill treating agents, CytoSol, a surface washing agent, and ThickSlick, a chemical herder, were evaluated based on their impacts on oil biodegradation. These two STAs drove changes in microbial community structure, potentially enriching for specific microbial groups that promoted complete compound mineralization to carbon dioxide. The biodegradation of certain classes of oil hydrocarbons was slowed by the application of these spill treating agents. However, the spill treating agents were easily broken down and consumed by the metabolically diverse oil-degrading microbial community. Due to their biodegradability, it is unlikely these products would negatively impact aerobic oil biodegradation in the marine environment.

## Data Availability

Sequences of 16S rRNA genes (DNA) and gene transcripts (RNA as cDNA) are publicly available in the National Center for Biotechnology Information (NCBI) Sequence Read Archive under accession number PRJNA1162750. Data are publicly available through the U.S. EPA ScienceHub website at https://doi.org/10.23719/1532068.

## SUPPLEMENTAL MATERIAL

Supplemental material aem.02334-24-s0001.pdf Supplemental methods, Figures S1 to S3, and Tables S1 to S3.

ASM does not own the copyrights to Supplemental Material that may be linked to, or accessed through, an article. The authors have granted ASM a non-exclusive, world-wide license to publish the Supplemental Material files. Please contact the corresponding author directly for reuse.
