# Supplementary material for: Oil spill surface washing agents and chemical herders drive microbial community structure impacting biodegradation
Source: Appl Environ Microbiol. 2025 Apr 22;91(5):e02334-24. doi: 10.1128/aem.02334-24 (PMC12093974; doi:10.1128/aem.02334-24)
Supplement: Supplemental material — Supplemental methods, Figures S1 to S3, and Tables S1 to S3. [file aem.02334-24-s0001.pdf]

Oil Spill Surface Washing Agents and Chemical Herders Drive Microbial Community Structure Impacting Biodegradation

Kiara L. Lech[a,#], Devi Sundaravadivelu[b], Robert J. Grosser[b], Leah R. Trutschel[c], Nichole E. Brinkman[a], Robyn N. Conmy[a]

[a]Office of Research and Development, Environmental Protection Agency, Cincinnati, Ohio, USA

[b]Pegasus Technical Services, Inc., Cincinnati, Ohio, USA

[c]Oak Ridge Associated Universities, Cincinnati, Ohio, USA

[#]Address correspondence to Kiara L. Lech, Email: Lech.Kiara@epa.gov

Supporting Information

MATERIALS AND METHODS

Headspace Analysis Using Gas Chromatography

A PorapakQ packed column (6´ × 1/8´´ ID; Supelco), 13X molecular sieve packed column, (10´ × 1/8´´ I.D.; Supelco), and automatic column valve switching enabled analyte separation and detection using a thermal conductivity detector (TCD) in-series with a methanizer/flame ionization detector (FID). Using a heated nickel catalyst, the methanizer converts carbon dioxide to methane before introduction into the FID, providing quantification of carbon dioxide at low concentrations. Injector, oven, TCD, and

FID temperatures were held constant at 200, 60, 150, and 350 °C, respectively. The GC was calibrated over a range of 1.0 to 20.9 % oxygen by volume using TCD and from 0.0875 % to 25 % carbon dioxide by volume using the methanizer/FID.

Total Organic Carbon

Samples were analyzed for TC by high-temperature oxidation and decomposition in a combustion furnace, followed by infrared (IR) detection of the carbon dioxide generated in the high temperature combustion process. A second aliquot of the sample was analyzed for IC content using acidification (ACS grade phosphoric acid, Fisher Scientific, Waltham, MA) and low-temperature heating to generate carbon dioxide from the carbonate component of the sample, followed by IR detection. The response from the detector was calibrated using ACS grade sucrose and sodium carbonate (Fisher Scientific, Waltham, MA) as respective TC and IC standards of known carbon composition, which were expressed as % carbon on a w/w basis. The total carbon (TC) fraction for sucrose (42.1 %, w/w basis) and the inorganic carbon (IC) fraction of sodium carbonate (11.3 %, w/w basis) were used to quantify Total Organic Carbon (TOC) in ANS and STAs (TC - IC = TOC, in percent basis, w/w).

Petroleum Hydrocarbons

To prepare the sample extracts for GS-MSD analysis, 90 µl of sample extract and 10 µl of 50 ppm internal standard mix were added to a salinized 200 µl insert and placed into a 2-ml autosampler vial with a Teflon lined cap. Standards were also prepared similarly by adding 90 µl of the standard mix (ranging 0.01 – 20 ppm) and 10 µl of 50 ppm internal standard mix. Vials were loaded onto the autosampler tray and analyzed in selected ion monitoring mode (SIM). One microliter of the standard or sample was injected in the splitless injection mode using helium as a carrier gas at a flow rate of 1 ml/min. The initial oven temperature was 50 °C, held for 1 min, followed by an increase to 325 °C at 10

°C/min, and was held at 325 °C for 8 min, with a total run time of 36.5 min. The MSD source and quadrupole were maintained at 300 °C and 200 °C, respectively.

Certified chemical standards for all target analytes, surrogates, and internal standards were purchased from various vendors detailed in Table S1. Standard solutions were prepared from pure standard materials or purchased certified mixes from 0.01 to 20 ppm. All solutions were prepared using methylene chloride (Optima™ grade, Fisher Scientific, Waltham, MA). The accuracy of the standards was verified against another certified source (Restek Corp., Bellefonte, PA; Cat. No. 31481). The method performance for PAHs and hopane detection was validated by analysis of a standard reference material, SRM 2779 – Gulf of Mexico Crude Oil (NIST, Gaithersburg, MD).

The target analytes included normal aliphatics ranging in carbon number from 10 to 35, branched alkanes (pristine and phytane), and fifty-two 2-6 ring PAH compounds and their alkylated homologs (including C0-4 – naphthalenes, C0-4 – phenanthrenes, C0-3 – fluorenes, C0-4 – dibenzothiophenes, C0-3 – naphthobenzothiophenes, C0-2 – pyrenes, C0-4 – chrysenes). For alkylated PAHs, the number of isomers increase significantly with increasing alkylation level and C1-C4 PAH isomers co-elute together with multiple peaks for the same ion. Therefore, it is not possible to identify all the alkylated isomers individually.[1, 2] Instead, for each level of alkylation, a representative PAH standard was selected to characterize the multiple isomers co-eluting in a sample (example: 2,3,5-Trimethylnaphthalene to represent all C3-naphthalenes) and the peaks were summed (Table S1). The non-alkylated parent or nearest neighbor compound was used to quantify the analyte incase standards are unavailable (example: C1-Chrysene was used to quantify C2-Chrysene). Additionally, 17α(H),21β(H)-Hopane was measured as a conservative biomarker.

For Total Petroleum Hydrocarbon (TPH) and Total Extractable Organic Matter (TEOM) analysis, 1 µl of the final extract was injected into the GC-FID, in the splitless injection mode, using helium as a

carrier gas at a 2 ml/min flow rate. The initial oven temperature was 50 °C, held for 2 min, followed by an increase to 300 °C at 30 °C/min, and held at 300 °C for 10 min, with a total run time of 20.33 min. The FID detector was operated at 320 °C with the hydrogen gas flow set at 40 ml/min and the airflow set at 450 ml/min. Chromatographic separation was performed using an Agilent DB-5MS column (30 m, 0.32 mm I.D., and 0.25 mm film thickness).

Droplet Digital Polymerase Chain Reaction (ddPCR)

Reactions were prepared in 22 µl volumes of 2X ddPCR Supermix for Probes (no dUTP, BioRad), 250 nM probe, 900 nM primers, and 5 µl of diluted sample. Droplets were made in the Automated Droplet Generator (BioRad) following the manufacturer's instructions. PCR was performed in a C1000 Touch Thermal Cycler (BioRad). Reactions were incubated at 95 °C for 5 min followed by 50 cycles of 95 °C for 30 sec and 60 °C for 1 min. The reaction was completed with a 98 °C incubation for 10 min. Amplification in droplets was assessed using the QX200 Droplet Reader (BioRad). Droplets were clustered using the AutoAnalyze (Combined Wells) function in QuantaSoft Analysis Pro (v.1.0596). Samples taken from replicate microcosms were analyzed by ddPCR in triplicate. Sample concentrations were determined by first calculating the number of molecules per droplet (λ) using the Poisson equation:

$$\lambda = \ln(1 - (\frac{p}{t})) \qquad (1)$$

where p is the number of positive droplets, and t is the total droplets.

Then, the following equation was applied to determine the number of 16S rRNA molecules (N) in the total volume of the extract:

$$N = \lambda(v \, * n) \left(\frac{x * E}{s * n}\right) d * \frac{1}{0.00085 \text{ µl}} \qquad (2)$$

where λ is the number of molecules per droplet, *v* is the ddPCR reaction volume (µl), *n* is the number of ddPCR reactions, *x* is the extract volume (µl), *E* is the number of extracts, *s* is the sample volume added to ddPCR reaction, *d* is the sample dilution factor, and 0.00085 represents the droplet volume in µl.

Quality control samples were included on each ddPCR plate to assess ddPCR performance. Negative control reactions consisted of 10 mM Tris-HCl (pH 8.5, Qiagen). Positive controls were derived from an engineered plasmid and were used as previously described.[3] Potential amplification inhibition in ddPCR reactions was monitored using a synthetic DNA molecule as previously described.[3] Inhibition was assessed by adding approximately 10^4 molecules of the inhibition control to each ddPCR reaction and comparing the results to sample-free reactions.

FIGURES AND TABLES

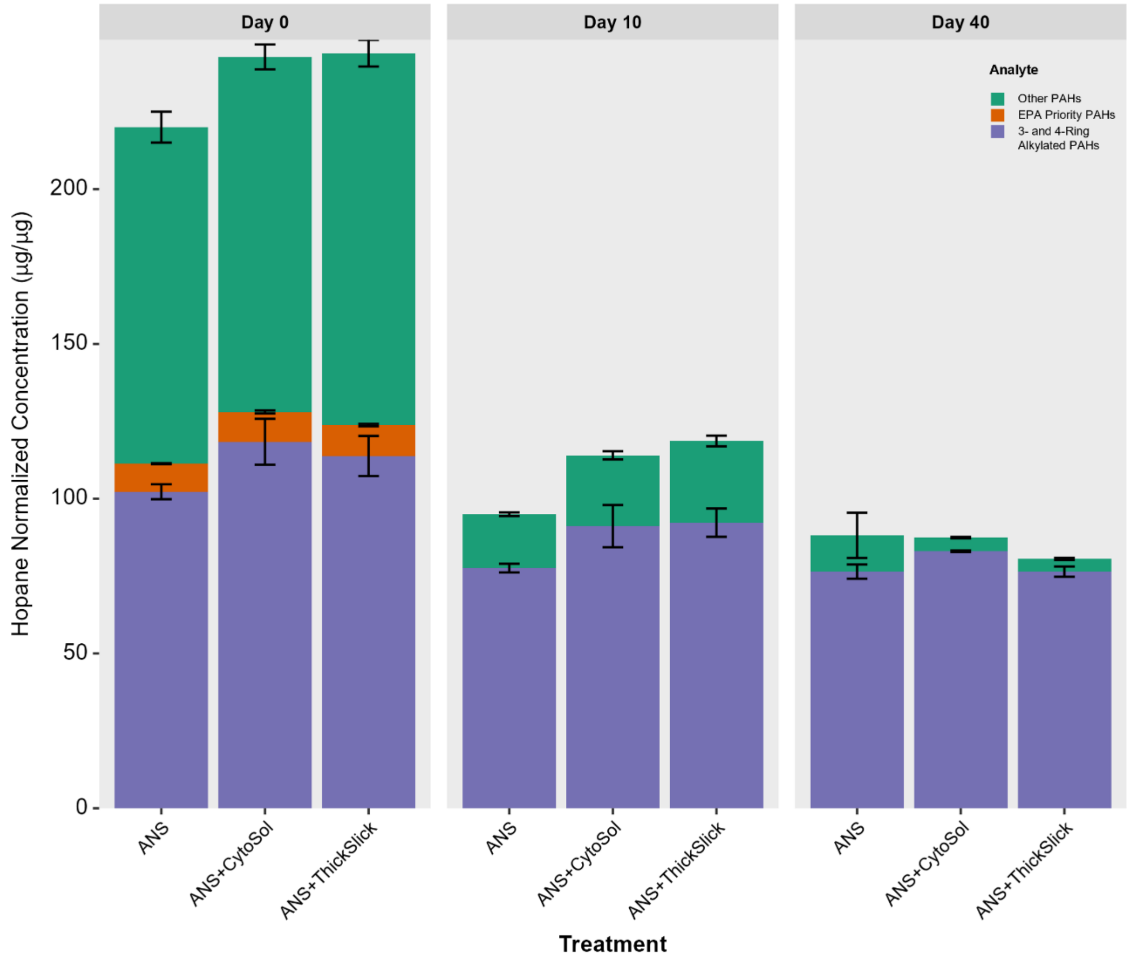

Figure S1: Concentrations of different classes of PAHs on select sampling days. Error bars represent ± 1 standard deviation of triplicate microcosms.

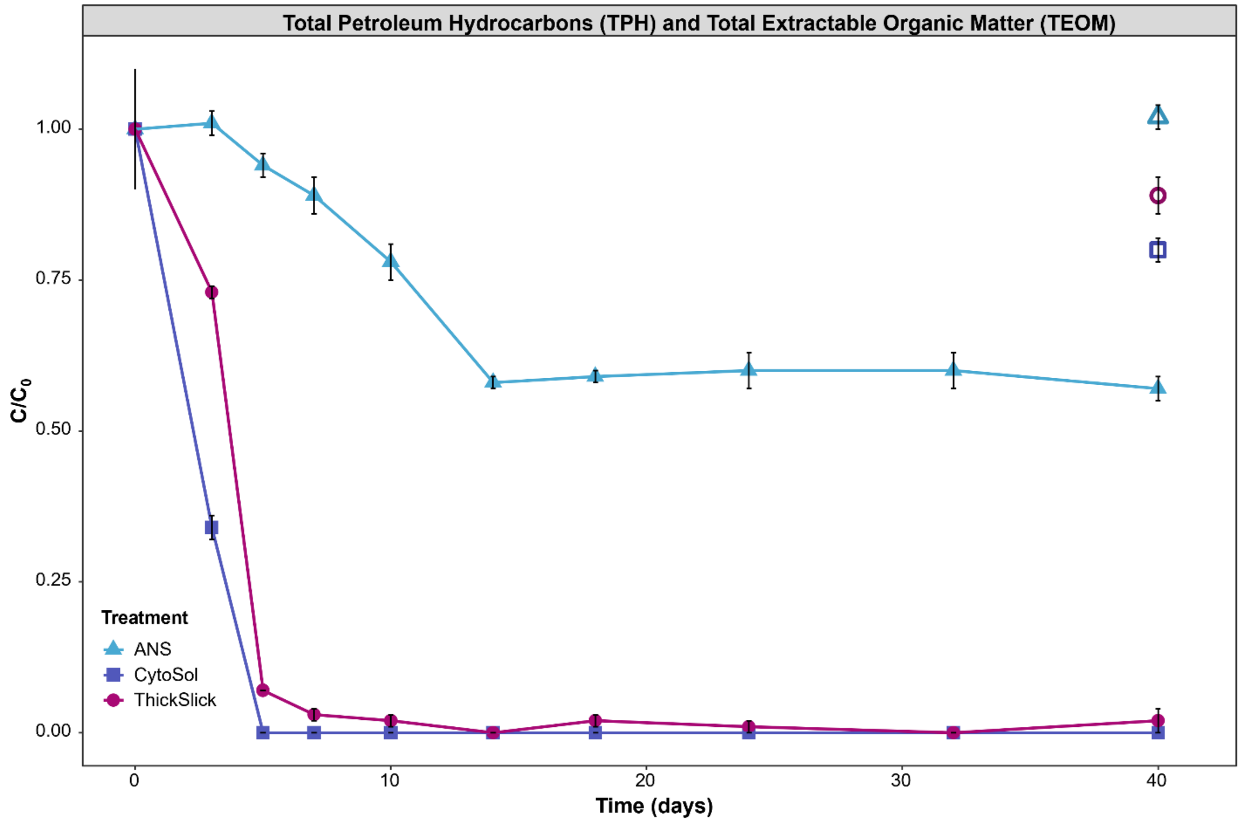

Figure S2: TPH and TEOM normalized to starting concentration in ANS and STA-only treatments (ThickSlick and CytoSol), respectively. Open symbols designate the killed controls corresponding to each treatment, sampled on Day 40. Error bars represent ± 1 standard error of triplicate microcosms.

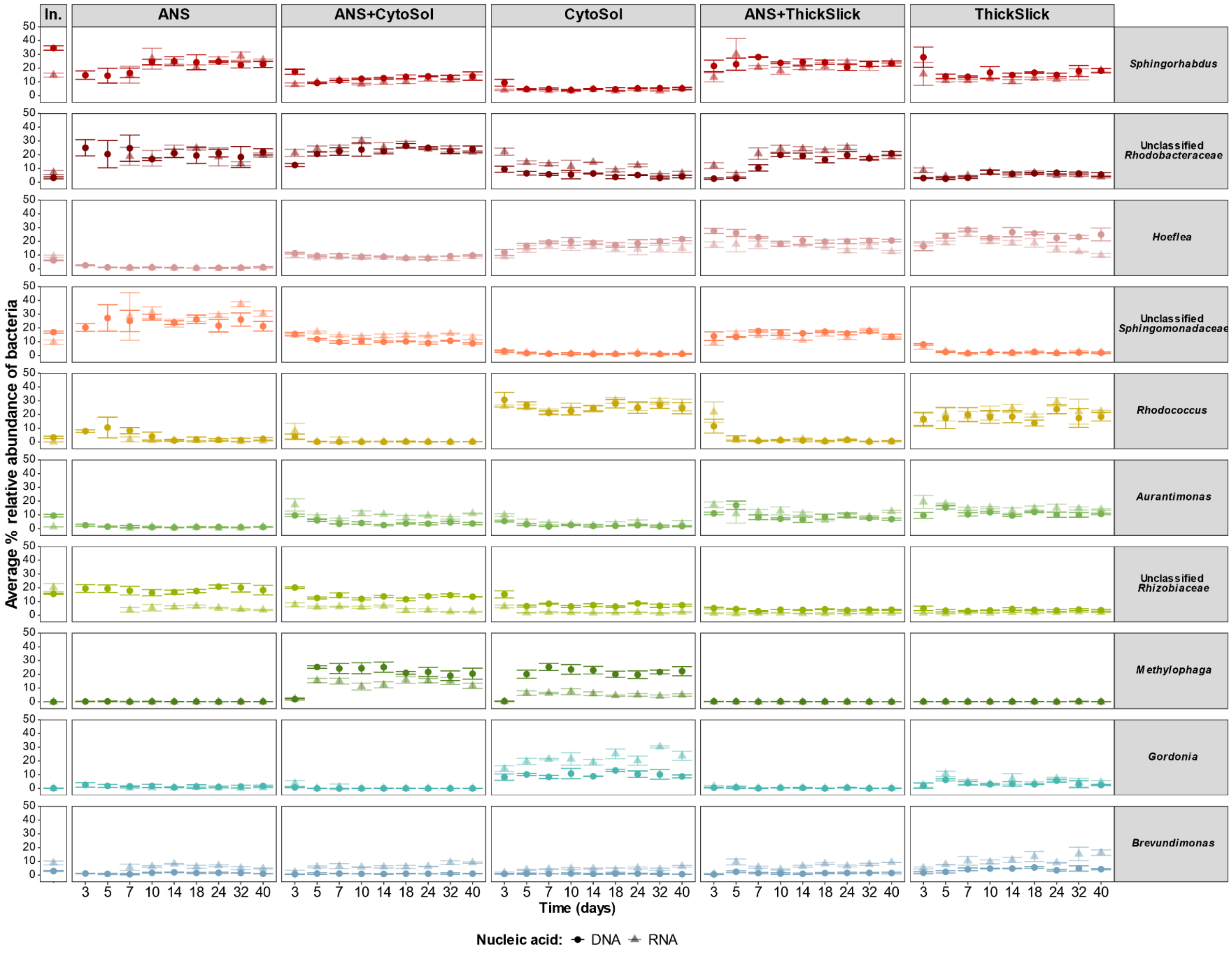

Figure S3: Dotplot showing change in relative abundance of the top ten genera over time and across treatments. Treatment "In." represents the initial inoculum used in the study. Error bars represent ± 1 standard deviation of triplicate samples. RNA samples are absent from ANS days 3 and 5 due to low yields during extraction.

Table S1. List of Chemicals Used for Alkanes and PAHs analysis.

Compound Name	Source/Catalogue Number
Internal Standards	
D22-n-Decane	Cambridge Isotopes, Cat. No. DLM-133-1
D34-n-Hexadecane	Cambridge Isotopes, Cat. No. DLM-203-1
D42-n-Eicosane	Cambridge Isotopes, Cat. No. DLM-2208-0.5
D62-n-Triacontane	Cambridge Isotopes, Cat. No. DLM-2210-0.5
D8-Naphthalene	Cambridge Isotopes, Cat. No. DLM-365-1
D10-Anthracene	Cambridge Isotopes, Cat. No. DLM-102-1
D12-Chrysene	Cambridge Isotopes, Cat. No. DLM-261-1
5α-Cholestane	Sigma Aldrich, Cat. No. C8003-1G
Surrogates	
D36-Heptadecane	Cambridge Isotopes, Cat. No. DLM-1342-1
D50-Tetracosane	Cambridge Isotopes, Cat. No. DLM-2209-0.5
D66-Dotriacontane	Cambridge Isotopes, Cat. No. DLM-2724-1
D10-1-Methylnaphthalene	Cambridge Isotopes, Cat. No. DLM-1607-1
D10-Phenanthrene	Cambridge Isotopes, Cat. No. DLM-371-1
D10-Pyrene	Cambridge Isotopes, Cat. No. DLM-155-0.5
5β-Cholestane	Santa Cruz Biotechnology, Cat. No. SC-214759
Target Analytes	
nc10	44 Compound Mix, Absolute Standards, Cat. No. 90311
nc11	44 Compound Mix, Absolute Standards, Cat. No. 90311
nc12	44 Compound Mix, Absolute Standards, Cat. No. 90311
nc13	44 Compound Mix, Absolute Standards, Cat. No. 90311
nc14	44 Compound Mix, Absolute Standards, Cat. No. 90311
nc15	44 Compound Mix, Absolute Standards, Cat. No. 90311
nc16	44 Compound Mix, Absolute Standards, Cat. No. 90311
nc17	44 Compound Mix, Absolute Standards, Cat. No. 90311
Pristane	44 Compound Mix, Absolute Standards, Cat. No. 90311
nc18	44 Compound Mix, Absolute Standards, Cat. No. 90311
Phytane	44 Compound Mix, Absolute Standards, Cat. No. 90311
nc19	44 Compound Mix, Absolute Standards, Cat. No. 90311
nc20	44 Compound Mix, Absolute Standards, Cat. No. 90311
nc21	44 Compound Mix, Absolute Standards, Cat. No. 90311
nc22	44 Compound Mix, Absolute Standards, Cat. No. 90311
nc23	44 Compound Mix, Absolute Standards, Cat. No. 90311
nc24	44 Compound Mix, Absolute Standards, Cat. No. 90311
nc25	44 Compound Mix, Absolute Standards, Cat. No. 90311
nc26	44 Compound Mix, Absolute Standards, Cat. No. 90311

nc27	44 Compound Mix, Absolute Standards, Cat. No. 90311
nc28	44 Compound Mix, Absolute Standards, Cat. No. 90311
nc29	44 Compound Mix, Absolute Standards, Cat. No. 90311
nc30	44 Compound Mix, Absolute Standards, Cat. No. 90311
nc31	44 Compound Mix, Absolute Standards, Cat. No. 90311
nc32	44 Compound Mix, Absolute Standards, Cat. No. 90311
nc33	44 Compound Mix, Absolute Standards, Cat. No. 90311
nc34	44 Compound Mix, Absolute Standards, Cat. No. 90311
nc35	44 Compound Mix, Absolute Standards, Cat. No. 90311
**Naphthalene**	44 Compound Mix, Absolute Standards, Cat. No. 90311
C1-naphthalene*	1-Methylnaphthalene in PAH 9 compound mix, Absolute Standards, Cat. No. 90822
C2-naphthalene*	2,6-Dimethylnaphthalene in PAH 9 compound mix, Absolute Standards, Cat. No. 90822
C3-naphthalene*	2,3,5-Trimethylnaphthalene in PAH 9 compound mix, Absolute Standards, Cat. No. 90822
C4-naphthalene*	1,4,6,7-Tetramethylnaphthalene, Toronto Research Chemical, Cat. No. T303670
**Acenaphthylene**	PAH 9 compound mix, Absolute Standards, Cat. No. 90822
Biphenyl	PAH 9 compound mix, Absolute Standards, Cat. No. 90822
**Acenaphthene**	PAH 9 compound mix, Absolute Standards, Cat. No. 90822
**Phenanthrene**	44 Compound Mix, Absolute Standards, Cat. No. 90311
**Anthracene**	44 Compound Mix, Absolute Standards, Cat. No. 90311
Dibenzofuran	Sigma-Aldrich, Cat. No. 45775-250MG
C1-phenanthrene*	2-Methylphenanthrene in PAH 9 compound mix, Absolute Standards, Cat. No. 90822
C2-phenanthrene*	3,6-Dimethylphenanthrene in PAH 9 compound mix, Absolute Standards, Cat. No. 90822
C3-phenanthrene*	1,2,6-Trimethylphenanthrene, Santa Cruz Biotechnology, Cat. No. SC-473002
C4-phenanthrene*	*Not Available (nearest neighbor C3-phenanthrene used to quantify)*
**Fluorene**	44 Compound Mix, Absolute Standards, Cat. No. 90311
C1-fluorene*	1-Methylfluorene, Sigma Aldrich, Cat. No. M46594-250MG
C2-fluorene*	1,8-Dimethylfluorene, Sigma Aldrich, Cat. No. S244678-25MG
C3-fluorene*	*Not Available (nearest neighbor C3-fluorene used to quantify)*
Dibenzothiophene	44 Compound Mix, Absolute Standards, Cat. No. 90311
C1-dibenzothiophene*	4-Methyldibenzothiophene, Sigma Aldrich, Cat. No. 496901-5G
C2-dibenzothiophene*	2,8-Dimethyldibenzothiophene, Toronto Research Chemical, Cat. No. D488280
C3-dibenzothiophene*	2,4,7-Trimethyldibenzothiophene, Santa Cruz Biotechnology, Cat. No. SC-474622

C4-dibenzothiophene*	*Not Available (nearest neighbor C3-dibenzothiophene used to quantify)*
Naphthobenzothiophene	1,2-Benzodiphenylene sulfide (or naphthobenzothiophene), Sigma-Aldrich, Cat. No. 255122-25MG
C1-naphthobenzothiophene*	*Not Available (nearest neighbor naphthobenzothiophene used to quantify)*
C2-naphthobenzothiophene*	*Not Available (nearest neighbor naphthobenzothiophene used to quantify)*
C3-naphthobenzothiophene*	*Not Available (nearest neighbor naphthobenzothiophene used to quantify)*
C4-naphthobenzothiophene*	*Not Available (nearest neighbor naphthobenzothiophene used to quantify)*
**Fluoranthene**	44 Compound Mix, Absolute Standards, Cat. No. 90311
2,3-Benzofluorene	2,3-Benzofluorene, Sigma-Aldrich, Cat. No. 123595-500MG
**Pyrene**	44 Compound Mix, Absolute Standards, Cat. No. 90311
C1-pyrene*	1-Methylpyrene, Sigma-Aldrich, Cat. No. 69025-100MG
C2-pyrene*	*Not Available (nearest neighbor C1-pyrene used to quantify)*
C3-pyrene*	*Not Available (nearest neighbor C1-pyrene used to quantify)*
C4-pyrene*	*Not Available (nearest neighbor C1-pyrene used to quantify)*
**Benzo(a)anthracene**	PAH 9 compound mix, Absolute Standards, Cat. No. 90822
Triphenylene	Triphenylene, Sigma-Aldrich, Cat. No. 45804-100MG
**Chrysene**	44 Compound Mix, Absolute Standards, Cat. No. 90311
C1-chrysene*	1-Methylchrysene, Toronto Research Chemical, Cat. No. M265115
C2-chrysene*	*Not Available (nearest neighbor C1-Chrysene used to quantify)*
C3-chrysene*	*Not Available (nearest neighbor C1-Chrysene used to quantify)*
C4-chrysene*	*Not Available (nearest neighbor C1-Chrysene used to quantify)*
**Benzo(b)fluoranthene**	44 Compound Mix, Absolute Standards, Cat. No. 90311
**Benzo(k)fluoranthene**	44 Compound Mix, Absolute Standards, Cat. No. 90311
Benzo(a)fluoranthene	Sigma-Aldrich, Cat. No. BCR097-100MG
Benzo(e)pyrene	44 Compound Mix, Absolute Standards, Cat. No. 90311
**Benzo(a)pyrene**	44 Compound Mix, Absolute Standards, Cat. No. 90311
Perylene	44 Compound Mix, Absolute Standards, Cat. No. 90311
**Indeno(1,2,3-cd)pyrene**	44 Compound Mix, Absolute Standards, Cat. No. 90311
**Dibenzo(a,h)anthracene**	44 Compound Mix, Absolute Standards, Cat. No. 90311
**Benzo(g,h,i)perylene**	44 Compound Mix, Absolute Standards, Cat. No. 90311
17α(H),21β(H)-Hopane	*Not Available (nearest neighbor 17β(H),21β(H)-Hopane, Supelco, Cat. No. 07562 used to quantify)*
** Summed peaks for particular alkylation level*<br>**Bold** *compounds are EPA high priority pollutant PAHs*	

Table S2: Pairwise Adonis comparisons of Bray-Curtis distances between treatment groups based on nucleic acid type. To account for multiple comparisons, the p value was adjusted using the Benjamini-Hochberg method.

Pairwise Adonis Comparison	F Statistic	R^2	Adjusted p value
**ANS RNA vs ANS DNA**	32.7	0.44	0.001
**ANS + CytoSol RNA vs ANS + CytoSol DNA**	37.5	0.42	0.001
**ANS + ThickSlick RNA vs ANS + ThickSlick DNA**	17.0	0.25	0.001
**CytoSol RNA vs CytoSol DNA**	54.5	0.51	0.001
**ThickSlick RNA vs ThickSlick DNA**	25.6	0.33	0.001

Table S3. Phylogenetic relationships among top ten most abundant genera in the treatments.

Kingdom	Phylum	Class	Family	Genus
Bacteria	Actinobacteriota	Actinobacteria	*Microbacteriaceae*	Unclassified *Microbacteriaceae*
			Nocardiaceae	*Rhodococcus*
				Gordonia
	Proteobacteria	Alphaproteobacteria	*Caulobacteraceae*	*Brevundimonas*
			Devosiaceae	*Pelagibacterium*
			Rhizobiaceae	*Hoeflea*
				Aurantimonas
				Unclassified *Rhizobiaceae*
			Rhodobacteraceae	Unclassified *Rhodobacteraceae*
			Sphingomonadaceae	*Sphingorhabdus*
				Unclassified *Sphingomonadaceae*
		Gammaproteobacteria	*Methylophagaceae*	*Methylophaga*

REFERENCES

1. Murray, J.; Sander, L.; Wise, S.; Reddy, C., Gulf of Mexico Research Initiative 2014/2015 Hydrocarbon Intercalibration Experiment: Description and Results for SRM 2779 Gulf of Mexico Crude Oil and Candidate SRM 2777 Weathered Gulf of Mexico Crude Oil. NIST Interagency/Internal Report (NISTIR), National Institute of Standards and Technology, Gaithersburg, MD: **2016**.
2. Yang, C.; Zhang, G.; Wang, Z. D.; Yang, Z. Y.; Hollebone, B.; Landriault, M.; Shah, K.; Brown, C. E., Development of a methodology for accurate quantitation of alkylated polycyclic aromatic hydrocarbons in petroleum and oil contaminated environmental samples. *Anal Methods-Uk* **2014,** *6* (19), 7760-7771.
3. Keely, S. P.; Brinkman, N. E.; Wheaton, E. A.; Jahne, M. A.; Siefring, S. D.; Varma, M.; Hill, R. A.; Leibowitz, S. G.; Martin, R. W.; Garland, J. L.; Haugland, R. A., Geospatial Patterns of Antimicrobial Resistance Genes in the US EPA National Rivers and Streams Assessment Survey. *Environ Sci Technol* **2022,** *56* (21), 14960-14971.
